# Continuous flexibility analysis of SARS-CoV-2 spike prefusion structures

**DOI:** 10.1107/S2052252520012725

**Published:** 2020-09-29

**Authors:** Roberto Melero, Carlos Oscar S. Sorzano, Brent Foster, José-Luis Vilas, Marta Martínez, Roberto Marabini, Erney Ramírez-Aportela, Ruben Sanchez-Garcia, David Herreros, Laura del Caño, Patricia Losana, Yunior C. Fonseca-Reyna, Pablo Conesa, Daniel Wrapp, Pablo Chacon, Jason S. McLellan, Hemant D. Tagare, Jose-Maria Carazo

**Affiliations:** a Centro Nacional de Biotecnologia–CSIC, Calle Darwin 3, 28049 Cantoblanco, Madrid, Spain; b Department of Radiology and Biomedical Imaging, Yale University, New Haven, CT 06520, USA; c Universidad Autónoma de Madrid, Calle Francisco Tomás y Valiente 11, 28049 Cantoblanco, Madrid, Spain; d Department of Molecular Biosciences, The University of Texas at Austin, Austin, TX 78712, USA; e Department of Biological Physical Chemistry, Instituto Rocasolano–CSIC, Calle de Serrano 119, 28006 Madrid, Spain

**Keywords:** cryo-electron microscopy, SARS-CoV-2, spike, conformational flexibility, image processing

## Abstract

Using a new consensus-based image-processing approach together with principal component analysis, the flexibility and conformational dynamics of the SARS-CoV-2 spike in the prefusion state have been analysed. These studies revealed concerted motions involving the receptor-binding domain (RBD), N-terminal domain, and subdomains 1 and 2 around the previously characterized 1-RBD-up state, which have been modeled as elastic deformations. It is shown that in this data set there are not well defined, stable spike conformations, but virtually a continuum of states. An ensemble map was obtained with minimum bias, from which the extremes of the change along the direction of maximal variance were modeled by flexible fitting. The results provide a warning of the potential image-processing classification instability of these complicated data sets, which has a direct impact on the interpretability of the results.

## Abstract 2

The flexibility and conformational dynamics of the SARS-CoV-2 spike in the prefusion state have been analyzed. An ensemble map with minimum bias was obtained, revealing concerted motions involving the receptor-binding domain (RBD), N-terminal domain and subdomains 1 and 2 around the previously characterized 1-RBD-up state, which have been modeled as elastic deformations.

## 1. Introduction   

SARS-CoV-2 infects target cells through the interaction of the viral spike (S) protein with cell receptors. This is an essentially dynamic event that is hard to analyze using most structural biology techniques. However, cryo-EM offers some unique capabilities that makes it a very suitable approach for this task, especially the facts that it can work with noncrystalline samples and, to a certain degree, those with structural flexibility (Dashti *et al.*, 2014 ▸; Maji *et al.*, 2020 ▸; Scheres *et al.*, 2007 ▸; Sorzano *et al.*, 2019 ▸; Tagare *et al.*, 2015 ▸).

In turn, cryo-EM information is complex, being buried in thousands of very noisy movies, making it a real challenge to reveal a three-dimensional (3D) structure from this collection of images. Furthermore, cryo-EM is in the middle of a methodo­logical and instrumental ‘revolution’ (Kühlbrandt, 2014 ▸) that has already been in progress for several years, with new methods constantly being produced. In this context, the original data of Wrapp *et al.* (2020 ▸) have been reanalyzed, applying newer workflows and algorithms, and thus obtaining improved information.

Considering that we were studying a biological system that is characterized by its continuous flexibility, we have not strictly followed the standard multi-class approach (Scheres *et al.*, 2007 ▸), which is very well suited to cases of discrete flexibility, since the mathematical modeling and the biological reality could be too far apart. Instead, we have calculated a new ‘ensemble’ map at 3 Å global resolution in which the bias has been carefully reduced, followed by both a 3D classification process and a continuous flexibility analysis in 3D principal component (PC) space using a GPU-accelerated and algorithmically improved version of the method of Tagare *et al.* (2015 ▸). The ensemble map was used for atomic modeling. Our aim was to explore a larger part of the structural flexibility present in the data set than is achievable by 3D classification alone. Using this mixed procedure, and through scatter plots of the projection of the different particle images onto the principal component axes, we have clearly shown how the spike flexibility in this data set should be understood as a continuum of states rather than discrete conformations. Using maximum-likelihood-based classification, we have obtained two maps that are projected at the extremes of the main principal component on which flexible fitting from the ensemble map has been performed. However, these extreme maps have an intrinsic blurring in the most flexible areas, since for any class that we may define the images come from a continuum of states and are therefore heterogeneous. This flexibility is substantially reduced in a recently described biochemically stabilized spike (Hsieh *et al.*, 2020 ▸), as shown by the reduced blurring, which translates into an improved local resolution.

In this work, we describe the new structural information that has been obtained and how it impacts our biological understanding of the system, together with the new workflows and algorithms that have made this accomplishment possible. We used *Scipion* 2.0 (de la Rosa-Trevín *et al.*, 2016 ▸) in order to easily combine different software suites in the analysis workflows. Maps and models have been deposited in public databases [EMPIAR (Iudin *et al.*, 2016 ▸) and EMDB (Lawson *et al.*, 2011 ▸)]: SARS-CoV-2 spike in the prefusion state as EMDB entry EMD-11328 and PDB entry 6zow, SARS-CoV-2 stabilized spike in the prefusion state (1-up conformation) as EMDB entry EMD-11341, SARS-CoV-2 spike in the prefusion state (flexibility analysis, 1-up closed conformation) as EMDB entry EMD-11336 and PDB entry 6zp5, and SARS-CoV-2 spike in the prefusion state (flexibility analysis, 1-up open conformation) as EMDB entry EMD-11337 and PDB entry 6zp7. All of the used data, the image-processing workflow and the intermediate results were also uploaded to EMPIAR (entries EMPIAR-10514 and EMPIAR-10516) by running the EMPIAR automatic deposition feature in *Scipion*.

## 2. Materials and methods   

### 2.1. Image-processing workflow   

The basic elements of the workflow combine classic cryo-EM algorithms with recent improvements in particle picking (Sanchez-Garcia *et al.*, 2018 ▸; Sanchez-Garcia, Segura *et al.*, 2020 ▸; Wagner *et al.*, 2019 ▸) and the key ideas of meta classifiers, which integrate multiple classifiers by a ‘consensus’ approach (Sorzano *et al.*, 2020 ▸), and finish with a totally new approach to map post-processing based on deep learning that we term *Deep cryo-EM Map Enhancer* (*DeepEMhancer*; Sanchez-Garcia, Gomez-Blanco *et al.*, 2020 ▸), which complements our previous proposal on local deblurring (Ramírez-Aportela, Vilas *et al.*, 2020 ▸). Naturally, map and map–model quality analyses are performed using a variety of tools (Pintilie *et al.*, 2020 ▸; Ramírez-Aportela, Maluenda *et al.*, 2020 ▸; Vilas *et al.*, 2020 ▸). Conformational variability analysis is carried out by explicitly addressing the continuously flexible nature of the underlying biological reality, in which the SARS-CoV-2 spike explores the conformational space to bind the cellular receptor. Most of the image processing performed in this work was performed using the *Scipion* framework (de la Rosa-Trevín *et al.*, 2016 ▸), which is a public domain image-processing framework that is freely available at http://scipion.i2pc.es.

A graphical representation of the image-processing workflow used in this work can be found in Supplementary Fig. S1.

### 2.2. Meta classifiers   

With meta classifiers, and as discussed in Sorzano *et al.* (2020 ▸), the rationale is that a careful analysis of the ratio between algorithmic degrees of freedom and data size shows that cryo-EM may has transitioned from an area characterized by parameter variance to one dominated by possible parameter biases. In very simple terms, we have a lot of data, so we can counteract the variance in our data if we deal with random errors. However, whenever there is the possibility of a systematic error, a so-called ‘bias’, artifacts may occur in the maps and, if this is the case, they can be very difficult to detect. We deal with the problem of introducing bias into the map through ‘consensus’, so that we select those parameters for which several methods, which are as methodologically ‘orthogonal’ as possible, concur on the same answer (sometimes we also use different runs of the same method).

This notion has been used in several different steps of the workflow as listed below.

(i) Contrast transfer function (CTF) estimation. We estimated the microscope defocus using two different programs: *Gctf* (Zhang, 2016 ▸) and *CTFFIND*4 (Rohou & Grigorieff, 2015 ▸). We only selected those micrographs for which both estimates agreed up to 2.1 Å resolution (Marabini *et al.*, 2015 ▸).
(ii) Particle selection. We used two particle-picking algorithms: *Xmipp* (Abrishami *et al.*, 2013 ▸) and *crYOLO* (Wagner *et al.*, 2019 ▸). We submitted both results to a picking consensus algorithm using deep learning (Sanchez-Garcia *et al.*, 2018 ▸) and also removed all of the coordinates in contaminations, carbon edges *etc.* using a deep-learning algorithm (Sanchez-Garcia, Segura *et al.*, 2020 ▸). We then cleaned the set of selected particles using two rounds of *cryoSPARC* 2D classification (Punjani *et al.*, 2017 ▸; Punjani & Fleet, 2020 ▸) and the consensus of two independent 3D classifications with *cryo­SPARC*.
(iii) Initial volume. As an initial volume, we selected the major class from the two 3D classifications above and refined it with *Xmipp Highres* (Sorzano *et al.*, 2018 ▸) with a local refinement of the 3D alignment.
(iv) 3D reconstruction. We then performed a *cryoSPARC* non-uniform 3D reconstruction, followed by a local angular refinement using *RELION* with a 3D mask (Zivanov *et al.*, 2018 ▸). Particle images were subjected to CTF refinement and Bayesian polishing (Zivanov *et al.*, 2018 ▸), before performing another two rounds of CTF refinement and local angular refinement in *RELION*, where we improved the resolution versus the first local refinement. Finally, we performed a non-uniform refinement in *cryoSPARC*. The reported nominal resolution of 2.96 Å is based on the gold-standard Fourier shell correlation (FSC) of 0.143 criterion. Actually, by using *Xmipp Highres* (Sorzano *et al.*, 2018 ▸) we could improve the resolution to 2.2 Å in the central region of the volume (the region that is not flexible), but at the expense of reducing it more in the flexible areas.
(v) 3D classification. We then performed two rounds of 3D classification with *RELION* followed by a consensus 3D classification, yielding two stables, large classes. Using these two classes, we then performed a local angular refinement using a *cryo­SPARC* non-uniform 3D reconstruction.

### 2.3. Particle selection   

We found that the micrographs and particles that are used for the 3D reconstruction play a key role in the quality and characteristics of the final map. In particular, we used the following two procedures.

(i) CTF estimation. We estimated the microscope defocus using *Gctf* and *CTFFIND*4. We required that both estimates were similar (the phase of their corresponding CTFs differed by less than 90°) up to 2.1 Å resolution. Only 70% of the micrographs met this criterion. We then estimated the CTF envelope using *Xmipp CTF* (Sorzano *et al.*, 2007 ▸) while keeping the defocus value fixed (calculated as the average of the *Gctf* and *CTFFIND*4 estimates). We found this step to be very important to retain high-resolution information. Using *Xmipp CTF*, we discovered that most of the micrographs had a non-astigmatic validity of between 3 and 4 Å (meaning that at this resolution the assumption of non-astigmatism broke down for most of the micrographs, and only a minority of 30% reached higher resolution in a non-astigmatic way).
(ii) Particle selection. Two advanced particle-picking algorithms were employed: *Xmipp* and *crYOLO*. The first identified 1.2 million coordinates possibly pointing to spike particles, while the second identified 730 000. We then combined the estimates using *Deep Consensus* with a threshold of 0.99, resulting in 620 000 coordinates. *MicrographCleaner* was used to rule out particles selected in the carbon edges, aggregations or contaminations, rejecting a total of 50 000 particles. After two rounds of *CryoSPARC* 2D classification with a pixel size of 2.1 Å and an image size of 140 × 140 pixels, we kept 298 000 particles assigned to 2D classes whose centroids clearly corresponded to projections of the spike. At this point, we performed two initial volume estimates using *CryoSPARC*, classifying the input particles into two classes. In both executions, one of the structures clearly corresponded to the spike (with 80% of particles), while the other resulted in a 3D structure that clearly corresponded to contamination. We calculated the consensus of the two *CryoSPARC* 3D classifications (those particles that were consistently assigned to the same 3D class). Only 203 000 particles belonged to the class that was consistently assigned to the spike.

### 2.4. Validation and quality analysis   

To judge the quality of our structural results, we concentrated here on three of the newest approaches: directional local resolution (Vilas *et al.*, 2020 ▸), *Q*-score (Pintilie *et al.*, 2020 ▸) and FSC-Q (Ramirez-Aportela, Maluenda *et al.*, 2020 ▸). The first provides a measure of map quality, while the latter two focus on the relationship between the map and the structural model; in other words, how well the model is supported by the map density, without any other complementary piece of information.

In terms of map-to-model validation, in Supplementary Figs. S3(*a*) and S3(*b*) we present *Q*-score and FSC-Q metrics, respectively, showing the agreement between the ensemble cryo-EM map and the structural model derived from it. In most areas the agreement is very good, with the exception of the receptor-binding domain (RBD) and substantial parts of the N-terminal domain (NTD), as expected from their higher flexibility.

### 2.5. Volume post-processing   

In this work, we used two volume post-processing approaches that both depart substantially from the traditional approach in the field, which is the application of global *B*-sharpening. One of the approaches is our previously introduced *LocalDeblur* sharpening method (Ramírez-Aportela, Maluenda *et al.*, 2020 ▸). The second approach is a totally new method based on deep learning (Sanchez-Garcia, Gomez-Blanco *et al.*, 2020 ▸). Concentrating on the latter, this method, *DeepEMhancer*, relies on a common approach in modern pattern recognition in which a convolutional neural network (CNN) is trained on a known data set comprised of pairs of data points and targets, with the aim of predicting the targets for new unseen data points. In this case, the training was performed by presenting the CNN with pairs of cryo-EM maps collected from the EMDB and maps derived from the structural models associated with the experimental maps. As a result, our CNN learned how to obtain much cleaner and detailed versions of the experimental cryo-EM maps, improving their interpretability.

Trying to take advantage of their complementary information, we used the two post-processed maps to trace the atomic model (PDB entry 6zow). Some examples of the similar improvement of structure modeling according to these two sharpened maps are shown in Supplementary Fig. S2. The sharpened and unsharpened maps have all been deposited in the EMDB.

### 2.6. Model building   

The atomic interpretation of the SARS-Cov-2 spike 3D map (PDB entry 6zow) was performed taking advantage of the modeling tools integrated in *Scipion* as described in Martínez *et al.* (2020 ▸). Owing to a lack of sufficient density for the ‘up’ conformation of the RBD, we rigidly fitted the structure of chain *A* (residues 336–525) of the SARS-CoV-2 RBD in complex with CR30022 Fab (PDB entry 6yla; J. Huo, Y. Zhao, J. Ren, D. Zhou, H. M. Ginn, E. E. Fry, R. Owens & D. I. Stuart, unpublished work) to the 3D map using *UCSF Chimera* (Pettersen *et al.*, 2004 ▸). This unmodeled part of the structure was called chain ‘*a*’ since it was part of chain *A* in the structure previously inferred from the same data set (PDB entry 6vsb; Wrapp *et al.*, 2020 ▸). The rest of the molecule was modeled using the same original structure (PDB entry 6vsb) as a template, as well as another spike ectodomain structure in the open state (PDB entry 6vyb; Walls *et al.*, 2020 ▸). The former structure (PDB entry 6vsb) was fitted to the new map and refined using *Coot* (Emsley *et al.*, 2010 ▸) and *phenix_real_space_refine* (Afonine *et al.*, 2018 ▸). Validation metrics were computed to assess the geometry of the new hybrid model and its correlation with the map using ‘Comprehensive Validation (cryo-EM)’ in *Phenix*, the *EMRinger* algorithm (Barad *et al.*, 2015 ▸), *Q*-score (Pintilie *et al.*, 2020 ▸) and FSC-Q (Ramírez-Aportela, Maluenda *et al.*, 2020 ▸). Score values considering the whole hybrid spike and excluding the unmodeled RBD are detailed in Supplementary Table S1. The hybrid atomic structures were submitted to the PDB.

*iMODFIT* (Lopéz-Blanco & Chacón, 2013 ▸) was employed to flexibly fit the hybrid atomic structure to the open and closed class maps.

### 2.7. Principal component analysis   

The principal component analysis used the expectation–maximization (EM) algorithm presented in Tagare *et al.* (2015 ▸) with the following minor modifications. Firstly, in contrast to Tagare *et al.* (2015 ▸), the images were not Wiener filtered, nor was the projected mean subtracted from the images; instead, the CTF of each image was incorporated into the projection operator of that image and a variable contrast was allowed for the mean volume in each image. The extent of the variable contrast was determined by the principal component EM algorithm. Secondly, the mean volume was projected along each projection direction and an image mask was constructed with a liberal soft margin to allow for heterogeneity. The different masks thus created, with one mask per projection direction, were applied to the images and the masked images were used as data. This step corresponds to imposing a form of sparsity on the data, which is known to improve the estimation of principal components in high-dimensional spaces (Johnstone & Paul, 2018 ▸). All images were downsampled by a factor of two to improve the signal-to-noise ratio and to speed up processing. Finally, during each EM iteration, the principal components were low-pass filtered with a very broad filter whose pass band extended to 4 Å. This helped with the convergence of the algorithm without significantly limiting the principal component resolution.

As part of the EM iteration, the algorithm in Tagare *et al.* (2015 ▸) conveniently estimates the expected amount by which each principal component is present in each image (this is the term E[z_j] in equation 15 of Tagare *et al.*, 2015 ▸). Fig. 3(*b*) shows a scatter plot of E[z_j].

It is interesting to note that in the algorithm of Tagare *et al.* (2015 ▸) the latent variables (representing the contributions of the principal components to each particle) are marginalized. Because of this marginalization, the number of unknown parameters that need to be estimated (the principal components and variances) is fixed and does not change with the number of particles. We have found this feature to be very valuable for relatively small sets of images (say 100 000 images), which is the case in our work, in order to prevent the number of parameters to be estimated growing with the number of particles. Statistically speaking, nonmarginalization is known to be a problem when there are few particles, where the estimates can be unreliable. Since the method developed by Tagare and coworkers does not suffer from this, we chose this method.

## 3. Results   

With the goal set at analyzing spike flexibility, we describe our key results step by step.

### 3.1. The ensemble map and the way to obtain it   

In the following, we describe the analysis of SARS-CoV-2 spike stabilized in the prefusion state by two proline substitutions in S2 (S-2P). We will objectively demonstrate that the flexibility of the spike protein should be understood as a quasi-continuum of conformations, so that when performing a structural analysis on this specimen special care has to be paid to the image-processing workflows, since they may directly impact on the interpretability of the results.

**Figure 1:**
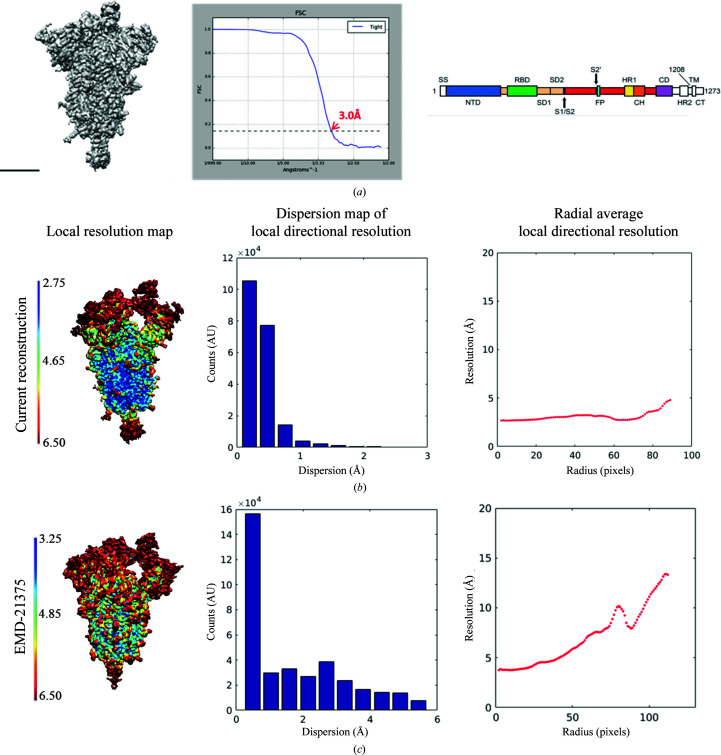
The spike and the ensemble map. (*a*) A representative view of the new map (EMDB entry EMD-11328), the corresponding FSC curve and the sequence of a monomer of the S protein (from Wrapp *et al.*, 2020). The scale bar is 5 nm in length. (*b*, *c*) New ensemble cryo-EM map (EMD-11328) compared with that originally presented (EMDB entry EMD-21375). The first row (*b*) corresponds to the new map and the second row (*c*) to EMD-21375. In each row, from left to right: a map representation showing the local resolution (computed with *MonoRes*; Vilas *et al.*, 2018 ▸), a histogram representation of the local directional resolution dispersion (interquartile range between percentiles 17 and 83) and, finally, a plot showing the radial average of the local tangential resolution (analyzed with *MonoDir*; Vilas *et al.*, 2020 ▸).

Starting from the original SARS-CoV-2 S-2P data set of Wrapp *et al.* (2020 ▸), we have completely reanalyzed the data using our public domain software integration platform *Scipion* (de la Rosa-Trevín *et al.*, 2016 ▸), breaking the global 3 Å resolution barrier. A representative view of the new ensemble map and its corresponding global FSC curve is shown in Fig. 1 ▸(*a*) (EMDB entry EMD-11328); the sequence of a monomer of the S protein is shown on the right to facilitate the further discussion of structure–function relationships (from Wrapp *et al.*, 2020 ▸). Figs. 1 ▸(*b*) and 1 ▸(*c*) show a comparison between the original map (Wrapp *et al.*, 2020 ▸) with EMDB code EMD-21375 and the newly reconstructed ensemble map corresponding to EMD-11328. Clearly, the local resolution (Vilas *et al.*, 2018 ▸), which is shown on the left in Figs. 1 ▸(*b*) and 1 ▸(*c*), is increased in the new map, and the anisotropy, which is shown in the center, is much reduced. Finally, on the right we present plots of the radially averaged tangential resolution, which is related to the quality of the angular alignment (Vilas *et al.*, 2020 ▸); the steeper the slope, the higher the angular assignment error. As can be appreciated, the slope calculated from the newly obtained map is almost zero when compared with that for the map from Wrapp *et al.* (2020 ▸), indicating that, in relative terms, the particle alignment used to create the new map is better than that used to build the original map. The result is an overall quantitative enhancement in map quality.

In terms of tracing, besides modeling several additional residue side chains and improving the geometry of the carbon skeleton (see Supplementary Fig. S2), one of the most noticeable improvements that we observed in the new map is an extension of the glycan chains that were initially built, particularly throughout the S2 fusion subunit (PDB entry 6zow). A quantitative comparison can be made between the length of glycan chains in the new ‘ensemble structure’ with respect to the previous structure (PDB entry 6vsb; see Supplementary Table S2). Although the total number of *N*-linked glycosylation sequons throughout the SARS-CoV-2 S trimer is essentially the same in the new structure (45) and PDB entry 6vsb (44), we have substantially increased the length of the glycan chains, expanding the total number of glycans by about 50%. We note the importance of this extensive glycosylation for epitope accessibility and how the accurate determination of this glycan shield will facilitate efforts to rapidly develop effective vaccines and therapeutics. Supplementary Fig. S2 shows a representative section of sharpened versions of the ensemble map (EMDB entry EMD-11328) compared with EMDB entry EMD-21375, in which the glycans can now be better traced. However, we should not forget that the ensemble map contains images in which the receptor-binding domain (RBD) and N-terminal domain (NTD) are in different positions (see Section 3.2), and consequently these domains appear to be blurred. Details of how the tracing was performed can be found in Section 2, while in Supplementary Fig. S3 we present two map-to-model quality figures indicating the good fit in general, with the obvious exception of the variable parts.

### 3.2. Flexibility analysis   

Starting from a carefully selected set of particles obtained from our consensus and cleaning approaches (see Section 2), together with the ensemble map described previously, we subjected the data to the following flexibility analysis.

**Figure 2:**
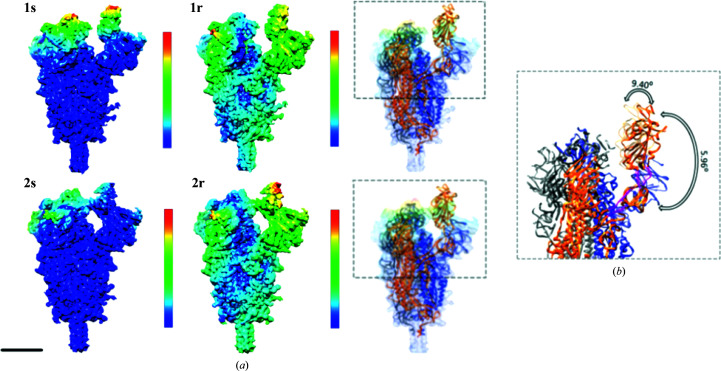
Flexibility analysis. (*a*) A representative view of the new ensemble map and the two new classes showing the ‘open conformation’ in Class 1 and the ‘closed conformation’ in Class 2. Note the elastic analysis of deformations performed on the Class 1 and Class 2 maps (see the main text), with 1s referring to ‘stretching’ and 1r to ‘rotations’. The color code is from blue for minimal deformation to red for maximal deformation. The scale bar is 5 nm in length. (*b*) Representation of the angles defined by the spike when transitioning between the opened and closed states. The regions shown in magenta represent the hinges used by the RBD domain to pivot. Note that each hinge encompasses two different chain regions. The first hinge spans amino acids 318–326 and 588–595, while the second hinge is defined by amino acids 330–335 and 527–531. The angles were measured using *PyMOL*.

The original images that were part of the ensemble map went through a ‘consensus classification’ procedure aimed at separating them into two algorithmically stable classes. Essentially, and as described in more detail in Section 2, we performed two independent classifications, further selecting those particles that were consistently together throughout the two classifications. In this way, we obtained the two new classes shown in Fig. 2 ▸(*a*). We will refer to these as the ‘closed conformation’ [Fig. 2 ▸(*a*), Class 1, EMDB entry EMD-11336] and the ‘open conformation’ [Fig. 2 ▸(*a*), Class 2, EMDB entry EMD-11337]. The number of images in each class was reduced to 45 000 in one case and 21 000 in the other, with global FSC-based resolutions of 3.1 and 3.3 Å, respectively.

The open and closed structures depict a clear and concerted movement of the ‘thumb’ formed by the RBD and sub­domains 1 and 2 (SD1 and SD2) and the NTD of an adjacent chain. The thumb moves away from the central spike axis, exposing the RBD in the up conformation. In order to make clearer where the changes are at the level of the Class 1 and Class 2 maps, we have made use of the representation of map local strains in Sorzano *et al.* (2016 ▸), which helps to very clearly visualize the type of strains needed to relate two maps, whether these are rigid-body rotations or more complex deformations (stretching). We have termed the maps resulting from this elastic analysis as ‘1s’ (Class 1, stretching) and ‘1r’ (Class 1, rotations) on the right-hand side of Fig. 2 ▸(*a*) and the same for Class 2. The color scale for both stretching and rotations goes from blue for small to red for large. Clearly, the differences among the classes with respect to the NTD and RBD have a very substantial component of pure coordinated rigid-body rotations, while the different RBDs present a much more complex pattern of deformations (stretching), indicating an important structural rearrangement in this area that does not occur elsewhere in the specimen. In terms of atomic modeling, we performed a flexible fitting of the ensemble model onto the closed and open forms [see Fig. 2 ▸(*a*), rightmost map; the PDB code for the open conformation is PDB entry 6zp7, while that for the closed conformation is 6zp5]. Focusing on rotations, which are the most simple element to follow, we can quantify that the degree of rotation of the thumb in these classes is close to 6°, as shown in Fig. 2 ▸(*b*). Given this flexibility, we consider that the best way to correctly present the experimental results is through the movie provided as Supplementary Movie S1, in which maps and atomic models are presented. Within the approximation to modeling that a flexible fitting represents, we can appreciate two hinge movements of the RBD–SD1–SD2 domains: one located between amino acids 318–326 and 588–595 that produces most of the displacement, and the other between amino acids 330–335 and 527–531 that accompanies a less pronounced ‘up’ movement of the RBD. This thumb motion is completed by the accompanying motion of the NTD from an adjacent chain. Also in a collective way, other NTDs and RBDs in the down conformation move slightly, as can better be appreciated in Supplementary Movie S1, where the transition between fitted models overlaps with the interpolation between observed high-resolution class maps.

**Figure 3:**
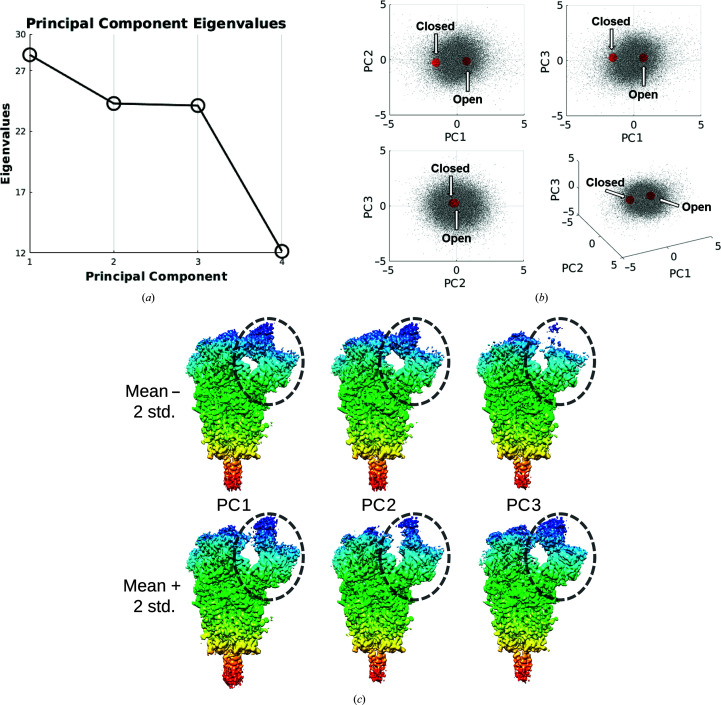
Principal component analysis of the SARS-CoV-2 spike structure. (*a*) Eigenvalues of principal components (PCs). The first three PCs are significant. (*b*) Scatter plot of the contribution of the first three PCs to each particle image together with the projection of the open and closed class maps, shown as red points. The difference between the projections of the two maps is mostly aligned along principal component 1 (PC1). (*c*) Side view of the first two PCs shown as mean ± 2 × std, where std is the square root of the eigenvalue. Coloring indicates the *z*-depth of the structure, and is added to assist visualization. Supplementary Figs. S4 and S5 contain additional views of these structures. The scale bar is 5 nm in length.

To further investigate whether or not the flexibility was continuous, we proceeded as follows. Images from the two classes were pooled together and, using the ensemble map, subjected to a 3D principal component analysis (PCA). The approach we followed is based on Tagare *et al.* (2015 ▸), with some minor modifications of the method. A detailed explanation of the modifications is given in Section 2. We initialized the first principal component (PC) to the difference between the open and closed conformation, while the remaining PCs were initialized randomly. Upon convergence, the eigenvalue of each PC and the scatter of the images in PC space was calculated. The eigenvalues of the PCs are shown in Fig. 3 ▸(*a*). Clearly, the first three PCs are significant. The scatter plot of the image data in PC1–PC3 space is shown in Fig. 3 ▸(*b*). Fig. 3 ▸(*b*) strongly suggests that there is ‘continuous flexibility’ rather than ‘tightly clustered’ flexibility. Fig. 3 ▸(*b*) also shows the projection of the maps corresponding to the open and closed conformations on the extremes of the first three PCs. It is clear that the open and closed conformations are aligned mostly along the first PC, suggesting that the open/closed classification captures the most significant changes. Fig. 3 ▸(*c*) shows side views of a pair of structures (mean ± 2 × std, where std is the square root of the eigenvalue) for each PC. Additional details of these structures are available in Supplementary Figs. S4 and S5. Note that PCs are not to be understood as structural pathways with a biological meaning, but as directions that summarize the variance of a data set. For instance, the fact that the RBD appears and disappears at the two extremes of PC3 indicates that there is an important variability in these voxels, which is probably indicative of the up and down conformations of the RBD [to be understood in the context of the elastic analysis shown in Fig. 2 ▸(*b*)].

Through this combination of approaches, we have learnt that the spike conformation fluctuates virtually randomly in a rather continuous manner. Additionally, the approach taken to define the two algorithmically stable ‘classes’ has clearly partitioned the data set according to the main axis of variance, PC1, since the projections of the maps of these classes fall almost exclusively along PC1 and are located towards the extremes of the image-projection cloud. Note that the fraction of structural flexibility owing to PC2 and PC3 is also important in terms of the total variance of the complete image set, but that classification approaches do not seem to properly explore it. Unfortunately, the resolution in PC2 and PC3 is currently limited, so it is difficult to derive clear structural conclusions from these low-resolution maps. However, it is clear from these data that the dynamics of the spike are far richer than just a rigid body closing and opening, and involves more profound rearrangements, especially at the RBD but also at other sites. This observation is similar to that of Ke *et al.* (2020 ▸) when working with subtomogram averaging.

Additionally, the fact that PCA indicates this continuous flexibility to be a key characteristic of the spike dynamics also suggests that many other forms of partitioning (rather than properly ‘classifying’) of this continuous data set could be devised, this fact just being a consequence of the intrinsic instability created by forcing a quasi-continuous data distribution without any clustering structure to fit into a defined set of clusters. In this work, we have clearly forced the classification to go to the extremes of the data distribution, as shown in Fig. 3 ▸, probably by enforcing an algorithmically stable classification, but the key result is that any other degree of movement of the spike in between these extremes of PC1 as well as PC2 and PC3 would also be consistent with the experimental data. In other words, since the continuum of conformations does not have clear ‘cutting/classification’ points, there is a certain algorithmic uncertainty and instability as to the possible results of a classification process. Note that this instability could be exacerbated by the step of particle picking, in the sense that different picking algorithms may have different biases (precisely to minimize this instability, we have performed a ‘consensus’ approach to picking throughout this work).

Clearly, flexibility is key in this system, so that alterations in its dynamics may cause profound effects, including viral neutralization, and this could be one of the reasons for the neutralization mechanism of antibodies directed against the NTD (Chi *et al.*, 2020 ▸).

### 3.3. Structure of a biochemically stabilized form of the spike   

**Figure 4:**
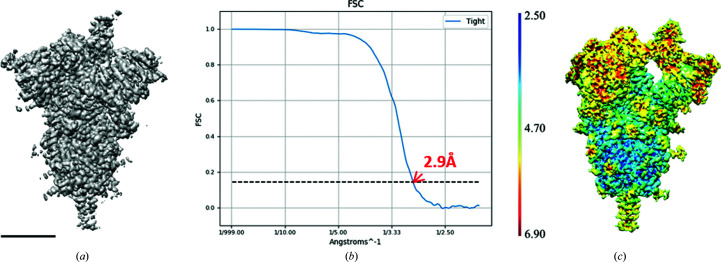
Analysis of a biochemically stabilized form of the spike. (*a*, *b*) A representative view of the stabilized form of the spike map and the corresponding FSC curve. The scale bar is 5 nm in length. (*c*) The local resolution map estimated with *MonoRes*.

We have also worked with a more recent variant containing six proline substitutions in S2 (HexaPro). This second protein was also studied by Hsieh *et al.* (2020 ▸). In this case, after going through the same stringent particle-selection process as for the previous specimen, as presented in depth in Section 2, it was impossible to obtain stable classes, so that in Fig. 4 ▸ we present a single map (EMDB entry EMD-11341) together with its global FSC curve and a local resolution analysis. It is clear that the local resolution has increased in the moving parts (mostly the RBD and NTD), although we did not feel confident in further modeling.

## 4. Conclusions   

In this work, we present a clear example of how the structural discovery process can be greatly accelerated by a wise combination of rapid data sharing and the use of the wave of newly developed algorithms that characterize this phase of the ‘cryo-EM revolution’. The reanalysis of the data used in Wrapp *et al.* (2020 ▸), but with new workflows and new tools, has resulted in a rich analysis of the spike flexibility as a key characteristic of the system.

Essentially, and at least to a first approximation, the spike moves in a continuous manner with no preferential states, as clearly shown in the scatter plots in Fig. 3 ▸(*b*). In this way, the result of a particular instance of image-processing analysis, including a 3D classification, should be regarded as a snapshot of this quasi-continuum of states. In our case, we have shown that a particular meta image-classification approach, implemented through a consensus among different methods in many steps of the analysis, results in classes that are at the extreme of the main axis of variance in PC space. Clearly, PC1, through the analysis of the two extreme classes, reflects a concerted motion of the NTD–RBD–SD1–SD2 thumb, although there are smaller collective movements throughout the spike (see Fig. 2 ▸ and Supplementary Movie S1). In this case, the RBD moves together with the NTD, with a smaller degree of independent flexibility and always in the ‘up’ conformation. The NTD–RBD movement can be characterized to a large degree as a rotation, but the different RBDs present a much more complex pattern of flexibility, indicating an important structural rearrangement [from elastic analysis (Fig. 2 ▸) and PCA (Fig. 3 ▸)]. The presence of quasi-solid body rotation hinges is clearly located between amino acids 318–326 and 588–595, which produce most of the displacement, together with other hinges between amino acids 330–335 and 527–531, which accompany a less pronounced ‘up’ movement of the RBD.

However, there are other PC axes explaining significant fractions of the inter-image variance that are not properly explored at the level of our two classes. PC3 is a clear example, indicating a high variance at the voxels associated with RBD up, which probably suggests large conformational changes in this area that result in the RBD moving down.

The flexibility analysis performed in this work complements previous analysis showing large rotations together with RBD up–down structural changes (Pinto *et al.*, 2020 ▸; Wrapp *et al.*, 2020 ▸), in the sense that the different studies present ‘snapshots’ of a continuum of movements obtained by a particular instance of an image-processing classification. In a sense, all of these results are correct, but none of them is able to capture the richness of the flexibility of this system. This fact reflects the intrinsic instability of segmenting a continuum into defined clusters, which is a clear limitation of the classification approaches that needs to be considered in detailed analysis of any data set from this system.

An obvious way to increase the resolution of the moving parts of the spike is to reduce their mobility, as is the case, for instance, in the biochemical stabilization of Hsieh *et al.* (2020 ▸) and also in the formation of a complex with an antibody against NTD (Chi *et al.*, 2020 ▸). On the other hand, the route towards a more complete analysis of the flexibility of the spike protein necessarily involves the analysis of data sets that are quite substantially larger than those being used in most current SARS-CoV-2 studies, so that all of the main axes of inter-image variability can be explored; this is work that is under development at the moment.

From a biomedical perspective, the proof that a quasi-continuum of flexibility is a key characteristic of this specimen, a concept that has been implicitly considered in much of the structural work performed so far but never demonstrated, suggests that ways to interfere with this flexibility could be important components of new therapies.

## Supplementary Material

EMDB reference: SARS-CoV-2 spike in prefusion state, EMD-11328

EMDB reference: SARS-CoV-2 spike in prefusion state (flexibility analysis, 1-up closed conformation), EMD-11336

EMDB reference: SARS-CoV-2 spike in prefusion state (flexibility analysis, 1-up open conformation), EMD-11337

EMDB reference: SARS-CoV-2 stabilized spike in prefusion state (1-up conformation), EMD-11341

PDB reference: SARS-CoV-2 spike in prefusion state, 6zow

PDB reference: SARS-CoV-2 spike in prefusion state (flexibility analysis, 1-up closed conformation), 6zp5

PDB reference: SARS-CoV-2 spike in prefusion state (flexibility analysis, 1-up open conformation), 6zp7

Supplementary Figures and Tables. DOI: 10.1107/S2052252520012725/fq5016sup1.pdf

Supplementary Movie S1. Movie presenting the morphing between the two algorithmically stable classes described in the main text, spanning principal component axis 1. DOI: 10.1107/S2052252520012725/fq5016sup2.mp4
