# Supplementary material for: Continuous flexibility analysis of SARS-CoV-2 spike prefusion structures
Source: IUCrJ. 2020 Sep 29;7(Pt 6):1059–69. doi: 10.1107/S2052252520012725 (PMC7553147; doi:10.1107/S2052252520012725)
Supplement: Supplementary file 1 [file m-07-01059-sup1.pdf]

# IUCrJ

**Volume 7 (2020)**


**Supporting information for article:**

## Continuous flexibility analysis of SARS-CoV-2 spike prefusion structures

**Roberto Melero, Carlos Oscar S. Sorzano, Brent Foster, José-Luis Vilas, Marta Martínez, Roberto Marabini, Erney Ramírez-Aportela, Ruben Sanchez-Garcia, David Herreros, Laura del Caño, Patricia Losana, Yunior C. Fonseca-Reyna, Pablo Conesa, Daniel Wrapp, Pablo Chacon, Jason S. McLellan, Hemant D. Tagare and Jose-Maria Carazo**

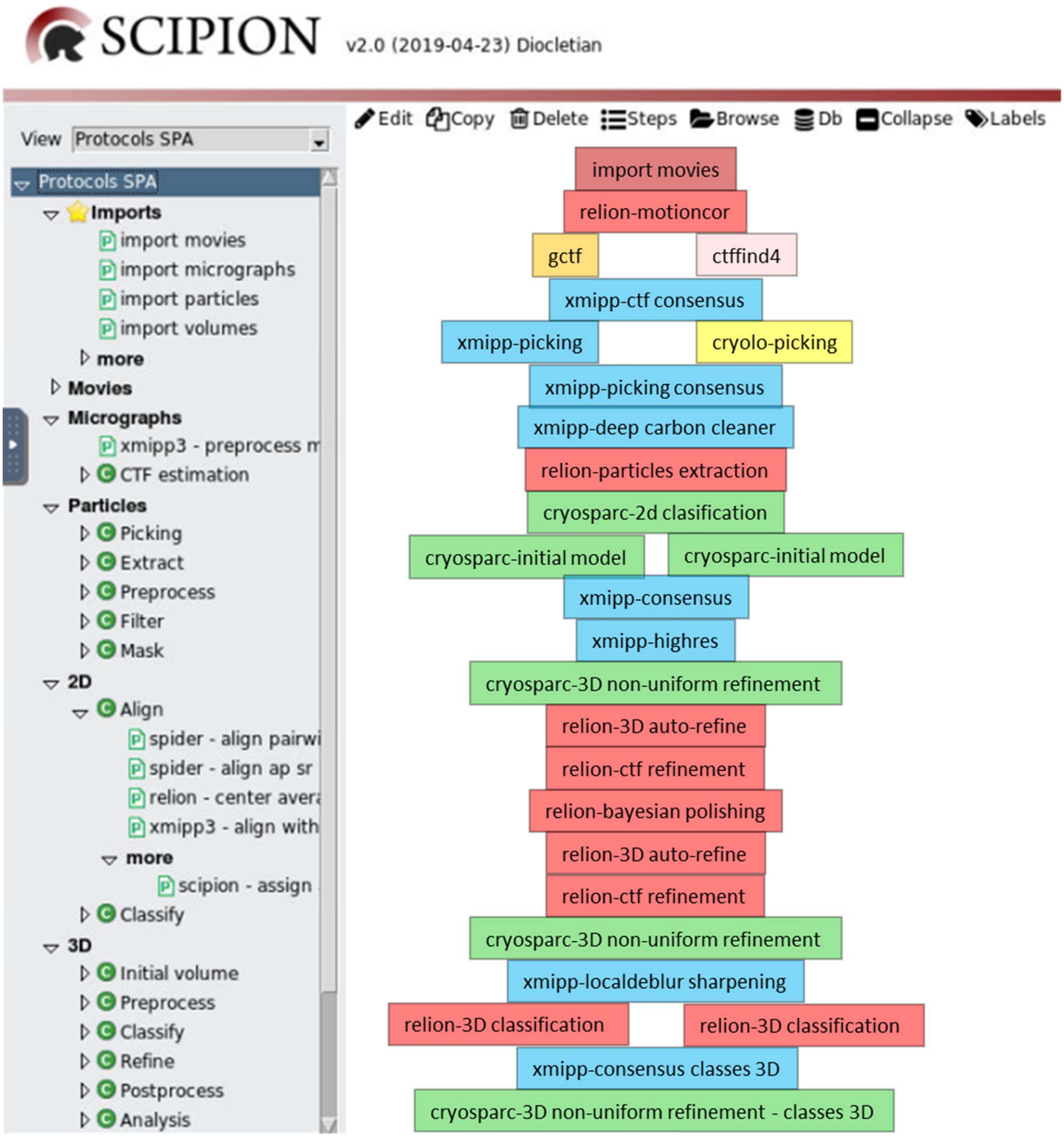


**Figure S1** Graphical representation of the processing workflow in Scipion. The workflow is also accessible at Scipion Workflow Repository at http://workflows.scipion.i2pc.es/.

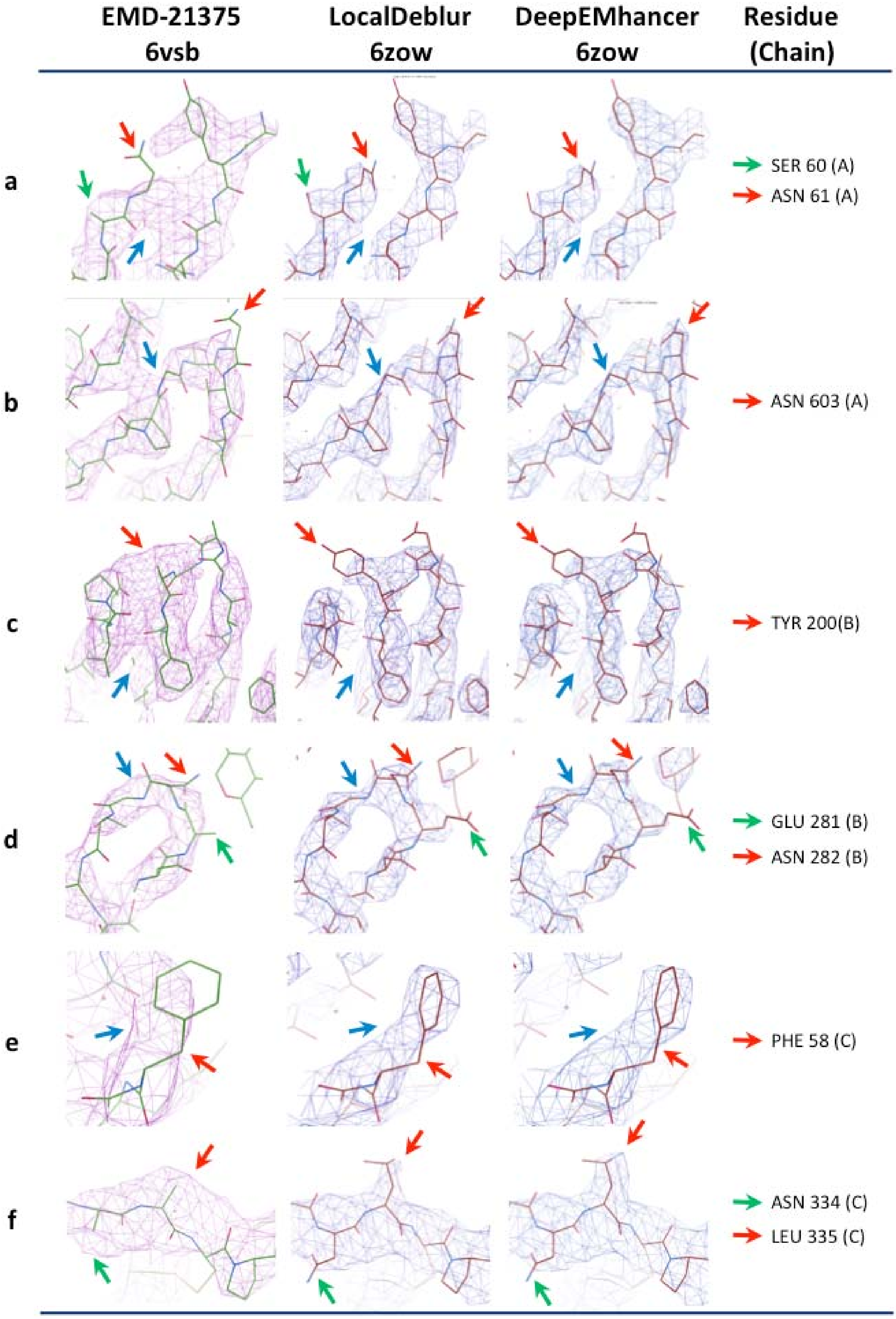

EMD-21375
6vsb
LocalDeblur
6zow
DeepEMhancer
6zow
Residue
(Chain)
a
SER 60 (A)
ASN 61 (A)
b
ASN 603 (A)
c
TYR 200(B)
d
GLU 281 (B)
ASN 282 (B)
e
PHE 58 (C)
f
ASN 334 (C)
LEU 335 (C)

**Figure S2** Comparison of the ability to trace the atomic structure between the original cryo-EM map (EMD-21375) and the two sharpened maps derived from the new reconstructed ensemble map. Six representative 3D map areas (a-f) illustrate the fitting between map and atomic structure. The red arrows detail amino acid side chains fitted to better defined densities in the sharpened map compared to the original map. These side chains could have been modeled (a, b, d, e) or being absent (c, f) in the original map. The green arrows indicate other additional residues whose side chains have been modeled only in the sharpened maps, while they were absent in the original one. The blue arrows point at densities that make it difficult to follow the carbon skeleton shape or discriminate among different chains in the original map, whereas they appear better resolved in the sharpened maps.

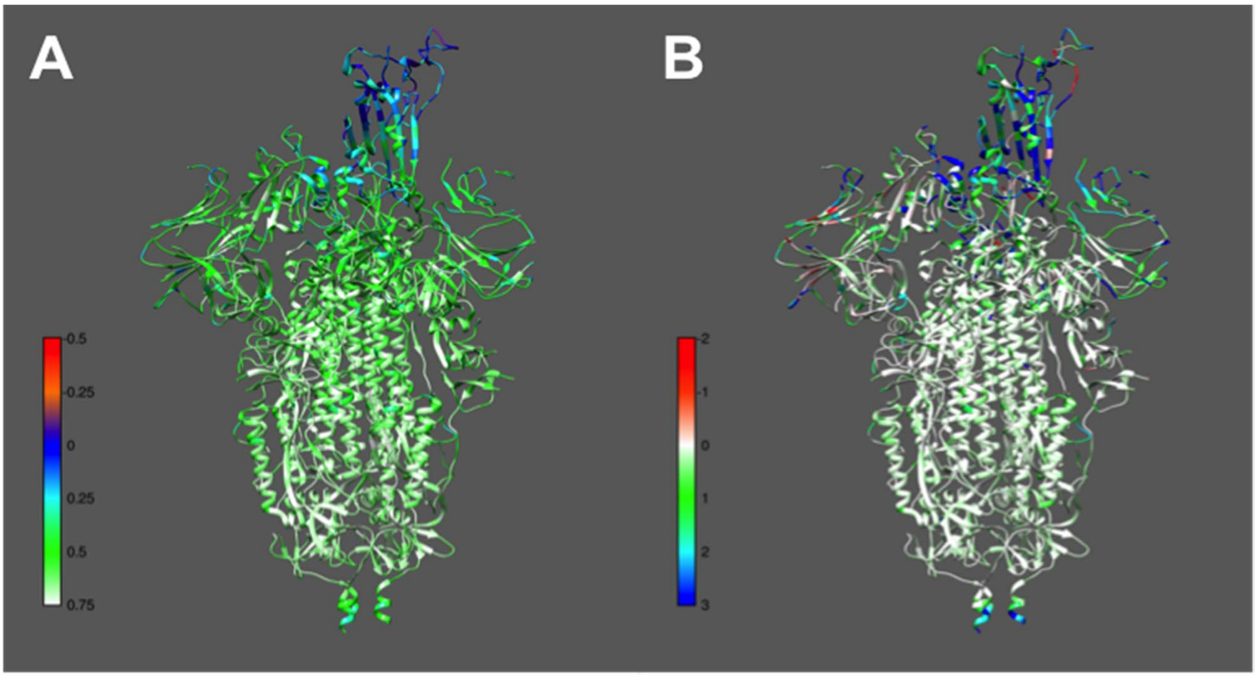


**Figure S3** Map-to-Model quality measures for the ensemble map. A) Q-score values represented on each amino acid of the new ensemble atomic model. Q-scores close to 1 indicate better resolvability of the residue. B) FSC-Q values are represented for each residue. Values close to zero indicate a good map-to-model fit, while values far from zero indicate areas where the model loses support with respect to the map signal.

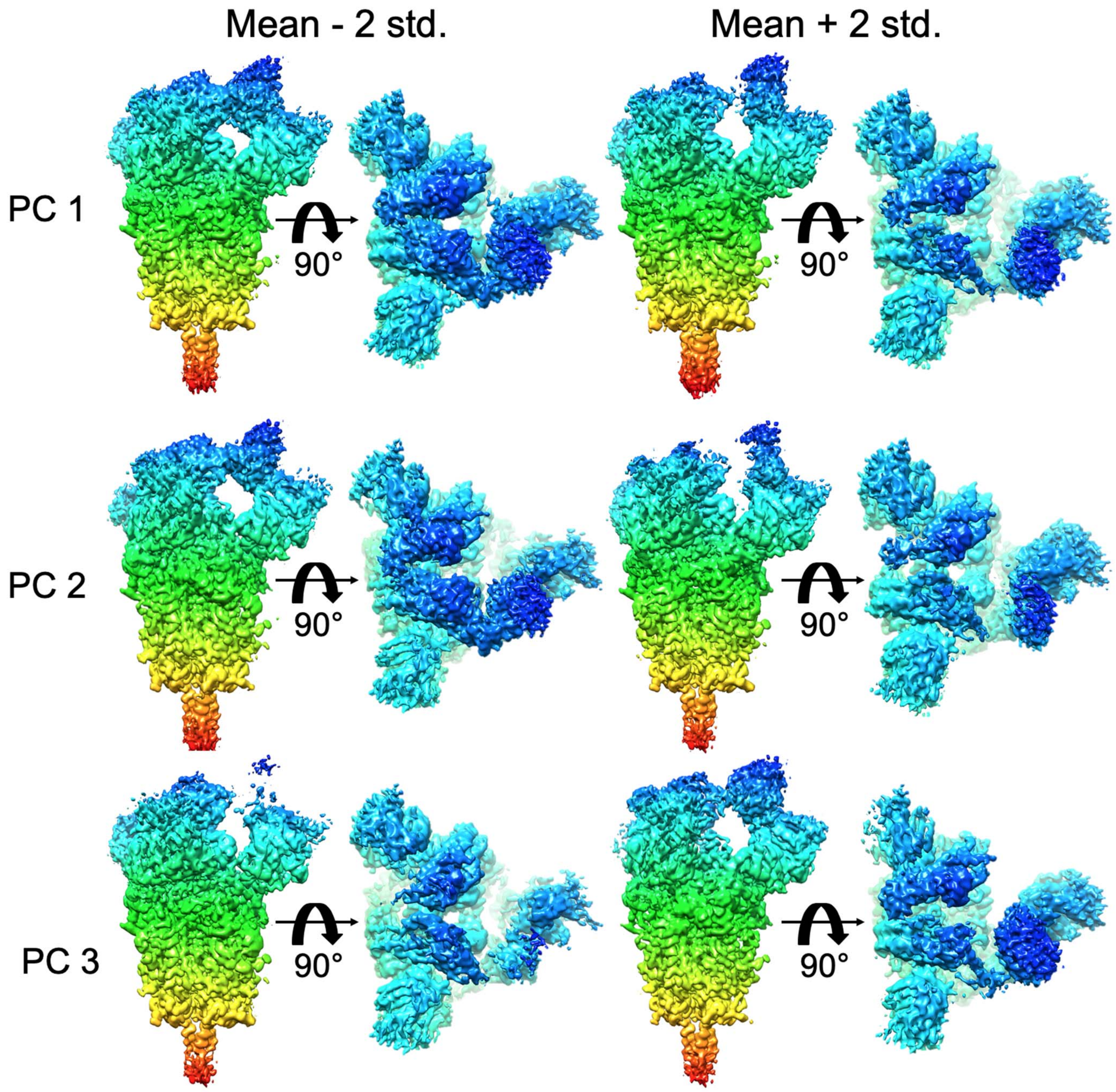


**Figure S4** Principal Component Analysis. Side and top views of mean volume +/- 2 std for the three principal components. Coloring indicates z-depth of the structure, and is added to assist visualization of the top view.

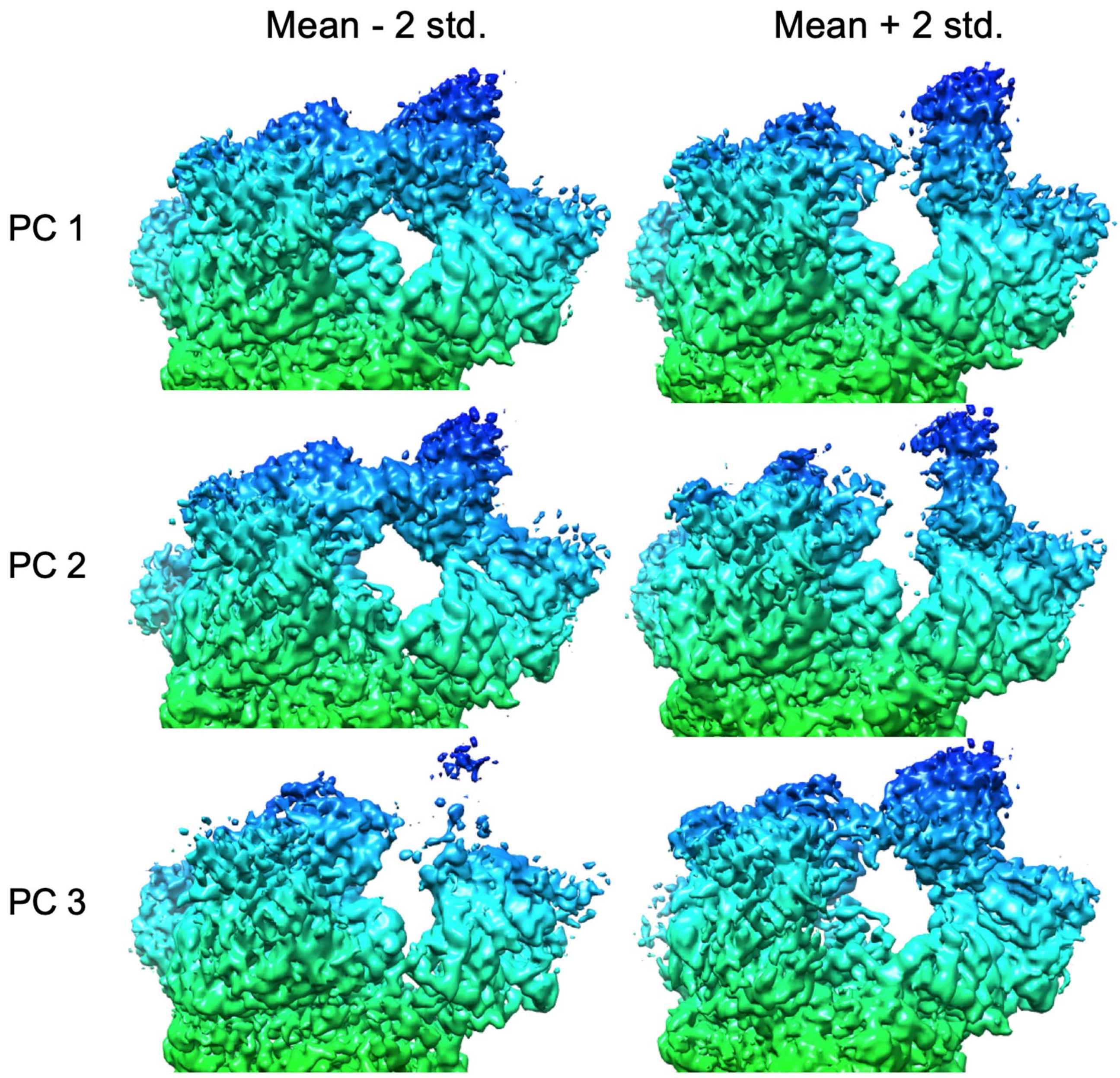


**Figure S5** Principal Component Analysis. Enlarged top half of the side views showing details of the density changes captured by the principal components. Coloring indicates z-depth of the structure, and is added to assist visualization of the top view.

**Table S1** Validation scores of the new atomic structure PDB 6ZOW

| Validation scores | Whole structure[1] | Modeled structure[2] |
|---|---|---|
| EMRinger | 2.66 | 2.73 |
| CC(mask) | 0.75 | 0.76 |
| Ramachandran outliers (Goal: < 0.2%) | 0.00 | 0.00 |
| Ramachandran favored (Goal: > 98%) | 97.06 | 97.28 |
| Rotamer outliers (Goal: < 1%) | 0.52 | 0.17 |
| C-beta outliers | 0 | 0 |
| Clashscore | 12.06 | 12.43 |
| MolProbity overall score | 1.76 | 1.74 |
| Q-Score | 0.55 | 0.58 |
| FSC-Q | 0.73 | 0.58 |

[1] Chains A, a, B, C

[2] Excluding the chain a (RBD, added by rigid fitting)

**Table S2** Number of sequons and size of their respective glycan chains.

| | | 6VSB | | 6ZOW | |
|---|---|---|---|---|---|
| Subunit | Size of N-linked glycan chain | Number of sequons | Number of glycans | Number of sequons | Number of glycans |
| S1 | 1 | 26 | 26 | 18 | 18 |
| | 2 | 0 | 0 | 7 | 14 |
| | 3 | 0 | 0 | 2 | 6 |
| | **Subtotal** | **26** | **26** | **27** | **38** |
| S2 | 1 | 1 | 1 | 0 | 0 |
| | 2 | 17 | 34 | 9 | 18 |
| | 3 | 0 | 0 | 5 | 15 |
| | 4 | 0 | 0 | 2 | 8 |
| | 5 | 0 | 0 | 2 | 10 |
| | **Subtotal** | **18** | **35** | **18** | **51** |
| **TOTAL** | | **44** | **61** | **45** | **89** |
